# Covert communication performance evaluation in UAV-assisted rate-splitting multiple access systems

**DOI:** 10.1371/journal.pone.0331013

**Published:** 2025-08-26

**Authors:** Nguyen Hong Nhu, Le Chi Bao, Bui Vu Minh, Minh Tran, Nguyen Quang Sang

**Affiliations:** 1 Faculty of Engineering and Technology, Saigon University, Ho Chi Minh City, Vietnam; 2 Transcosmos Vietnam, Ho Chi Minh City, Vietnam; 3 Faculty of Engineering and Technology, Nguyen Tat Thanh University, Ho Chi Minh City, Vietnam; 4 Advanced Intelligent Technology Research Group, Faculty of Electrical and Electronics Engineering, Ton Duc Thang University, Ho Chi Minh City, Vietnam; 5 Posts and Telecommunications Institute of Technology, Ho Chi Minh City, Vietnam; Beijing Institute of Technology, CHINA

## Abstract

In this paper, we investigate using rate-splitting multiple access (RSMA) to facilitate covert communication in a multi-user unmanned aerial vehicle (UAV) downlink communication network that is being monitored by a warden (Willie). We establish a comprehensive analytical framework and derive closed-form expressions for key performance metrics under Nakagami-m fading channels. Specifically, we analyze the detection error probability (DEP) at Willie to quantify system covertness, in addition to outage probability (OP) and ergodic rate (ER) experienced by legitimate users, along with asymptotic analysis in the high signal-to-noise ratio (SNR) region. Furthermore, we propose an efficient alternating optimization algorithm to determine the optimal static position of the UAV that maximizes system covertness. Numerical simulations support the theoretical results derived, present the impact of various system parameters, and provide a performance comparison with non-orthogonal multiple access (NOMA). Results indicate that RSMA offers significant covertness gains over the NOMA scheme.

## 1 Introduction

Unmanned Aerial Vehicle (UAV) communications play a vital role in enabling seamless and reliable data exchange between drones and their operators or other connected devices. These communication systems are essential for controlling the UAVs [1], transmitting real-time data, and ensuring safe and efficient operations in various industries [2]. UAV communications refer to the technologies, protocols, and systems that enable UAVs to exchange information with ground stations, other UAVs, and network infrastructure [3,4]. Moreover, UAV will be a promising platform for future wireless networks because it can provide rapid deployment, flexible mobility, and line-of-sight (LoS) control of ground terminals [5–7]. All these characteristics render UAVs highly desirable for applications with increased coverage or ad hoc infrastructure, e.g., disaster recovery, remote sensing, and Internet of Things (IoT) coverage [8,9]. However, the broadcast nature of UAV-to-ground communications also exposes them to eavesdropping and detection by unauthorized wardens in physical layer security (PLS). This raises serious concerns for applications that demand confidentiality or stealth [10,11].

PLS in communication systems is a set of techniques and principles designed to protect wireless communications by leveraging the inherent characteristics of the physical communication medium, rather than relying solely on traditional cryptographic methods [12]. PLS exploits properties such as channel randomness, noise, and multipath propagation to ensure that confidential information remains secure, even in the presence of eavesdroppers or malicious attacks [13–15]. Covert communication represents an advanced form of PLS that aims not only to protect the content of transmissions but also to hide the very existence of communication[16], sometimes, referred to as low-probability-of-detection (LPD) communication, aims to hide the very existence of a transmission from a warden [17,18]. Unlike classical PLS that assumes the warden is oblivious to the transmission time or scheduling, covert schemes must ensure that any statistical test performed by the warden cannot reliably distinguish between the presence and absence of a covert signal [19,20]. Recent works have analyzed fundamental limits of covert throughput over additive white Gaussian noise (AWGN) and fading channels, revealing the so-called “square-root law” which constrains the number of reliably covert bits to scale only on the order of the square root of the blocklength [21–23]. Extending these insights to more complex network and channel models, including relay-assisted, large-scale, and reconfigurable intelligent surface (RIS) setups, has attracted significant interest [24,25].

Meanwhile, Rate-Splitting Multiple Access (RSMA) has emerged as a transformative interference management and multiple access framework for 6G networks [26], combining the benefits of both non-orthogonal and orthogonal transmission strategies. In addition, RSMA has gained prominence for its ability to flexibly manage multi-user interference and improve spectral and energy efficiency in downlink systems [27–29]. By intelligently splitting user messages and managing interference, RSMA achieves superior spectral efficiency compared to conventional 5G techniques like space-division multiple access (SDMA) and non-orthogonal multiple access (NOMA) [30,31]. Recently, the studies have demonstrated RSMA’s benefits for PLS, where the common stream can serve as artificial noise to confuse eavesdroppers, and for covert communications in terrestrial multi-antenna networks [32,33]. Combining RSMA and covert communication represents an advanced approach to achieving both spectral efficiency and undetectable transmissions in next-generation wireless networks.

Despite these advances, the integration of RSMA with UAV-enabled covert communications remains largely unexplored. The mobile and altitude-dependent channel characteristics of UAV links introduce new degrees of freedom for covertness, but also complicate the warden’s detection strategy [34]. Moreover, while NOMA-based covert UAV schemes have been studied [35], their performance is fundamentally limited by the rigid power domain multiplexing, which may leave covert signals more vulnerable to detection. From a covert communication perspective, NOMA’s fixed decoding order and predetermined power levels constrain the transmitter’s flexibility, making covert signals easier to isolate and detect. RSMA, on the other hand, provides a more agile structure through the decomposition of messages into public (common) and private parts. This stream splitting not only allows finer control over power allocation but also enables covert signals to be embedded dynamically in the private stream, while masking them under the common layer. Such flexibility strengthens the system’s ability to regulate covertness under diverse channel conditions, making RSMA a more robust solution for secure and stealthy UAV communication. By contrast, RSMA offers a richer design space through joint power-splitting and spatial parameters, potentially leading to improved covertness without sacrificing user reliability.

### 1.1 Related work

**Covert UAV Communications:** Prior works on UAV covert communications have considered scenarios with cooperative jamming [36], trajectory optimization [37], and energy harvesting [38]. These studies typically adopt NOMA or orthogonal multiple access (OMA) and focus on maximizing the covert throughput or minimizing the warden’s detection probability under flight-path constraints. However, they do not exploit the message-splitting flexibility of RSMA.

**RSMA in Secure and Covert Systems:** In terrestrial networks, RSMA has been applied to enhance secrecy rates against passive eavesdroppers by judiciously allocating power to the common stream as artificial noise [39], and to improve covert performance in multi-user MISO setups by optimizing the power split between common and private messages [40]. These results suggest that RSMA could similarly benefit UAV systems, but the unique air-to-ground fading and path-loss characteristics necessitate a dedicated analysis. In the context of covert multi-user transmission, the work in [41] analyzes covert capacity achieved when overt users are randomly activated, providing theoretical benchmarks that align with our interest in user-oriented covert strategies. Moreover, the Delay-Doppler Domain SSMA framework for satellite networks introduced in [42] presents a novel signal separation approach across multiple branches, which shares conceptual similarities with RSMA’s layered message splitting. These references highlight alternative architectures and underline the growing interest in secure, flexible access schemes, although our system and objectives differ significantly.

### 1.2 Motivation and contributions

Motivated by the need to bolster covert UAV communications in the presence of a vigilant warden, this paper develops and analyzes an RSMA-based downlink scheme over Nakagami-*m* fading channels. Our main contributions are as follows:

- We propose an RSMA-based framework for covert UAV-downlink communication under Nakagami-*m* fading and formulate a general analytical model that encompasses both the warden’s detection error probability (DEP) and legitimate users’ performance.
- We derive in closed form the DEP at Willie, the OP of every user, and the ER, and offer high-SNR asymptotic expressions where the system’s diversity order and rate ceilings are considered. We also conduct large-scale Monte Carlo simulations to confirm our theoretical analysis, investigating the impact of the critical system parameters, power allocation, UAV altitude, fading severity, and detection threshold on covertness and reliability.
- Finally, we contrast RSMA to a baseline NOMA system and demonstrate that, through dynamic power allocation to common and private streams, RSMA yields significantly better minimum DEP (i.e., secrecy) with the same OP and ER performance.

### 1.3 Organization

The rest of this paper is structured as follows. Sect 2 describes the system, channel model, and RSMA transmission protocol. Sect 3.1 provides the closed-form analysis of Willie’s detection error probability. Sect 3.2 introduces an optimization algorithm for the UAV’s position. Sect 3.3 develops the outage probability of legitimate users, and Sect 3.4 develops ergodic rate expressions and their behavior in the high-SNR regime. Sect 4 confirms all analytical findings by numerical simulations and compares RSMA and NOMA in different setups. Sect 5 concludes the paper and presents some open directions for future research.

## 2 System model and communication protocol

### 2.1 System and channel model

**Fig 1:**
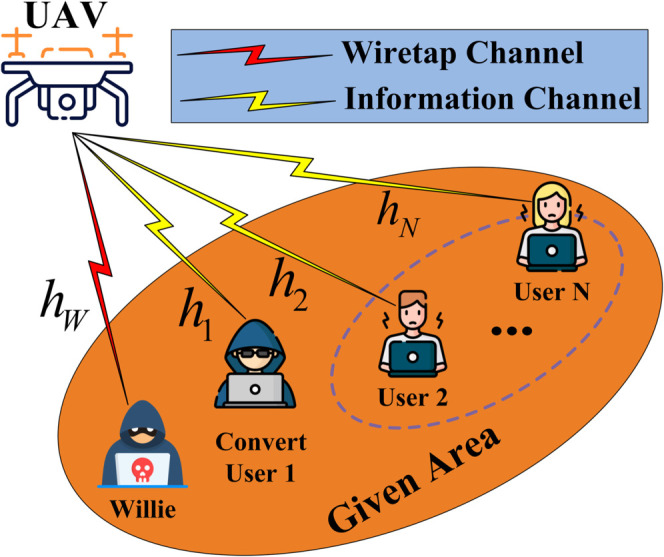
The system model of UAV-enabled RSMA network.

In this section, we introduce a model for a covert communication framework grounded in RSMA, illustrated in Fig 1. This framework comprises a UAV, *N* users including a covert user (User 1) and a set of public users (User 2,..., User *N*), along with a warden (Willie). Each terminal device incorporated within the system is outfitted with a single antenna. We assume that all channels are characterized by independent Nakagami-*m* fading, with *h*_*i*_ and *h*_*W*_ representing the instantaneous channel fading coefficients from the UAV to the *i*th user (User *i*) and Willie, respectively. The terms ${\left|h_{i}\right|}^{2}$ and ${\left|h_{W}\right|}^{2}$ denote the instantaneous gain in the channel from the transmitting UAV to the receiving *i*th user and from the UAV to Willie, respectively. An enumeration of the critical symbols is provided in Table 1.

**Table 1:** List of key symbols.

Symbol	Description
*N*	Number of users
$r_{i},r_{W}$	Horizontal distances UAV-User *i* and UAV-Willie
$d_{i},d_{W}$	Euclidean distances UAV-User *i* and UAV-Willie
$\varphi_{a}$	Elevation angle between UAV and terminal *a* (a∈{i,W})
*δ*	Path-loss exponent
α,β	Environment-dependent constants in LoS/NLoS model
$N_{\text{LoS}},\;N_{\text{NLoS}}$	Additional path-loss factors for LoS and NLoS
*f*_*c*_	Carrier frequency
*c*	Speed of light
$|h_{i}{|}^{2},\;|h_{W}{|}^{2}$	Instantaneous channel power gains to user *i* and Willie
$m_{i},\;m_{W}$	Nakagami-*m* fading severity factors for user *i* and Willie
$\Omega_{i},\;\Omega_{W}$	Average channel power gains for user *i* and Willie
*a*_*c*_	Power allocation coefficient for the common message stream
*a*_*i*_	Power allocation coefficient for *i*th user private message
$a_{p}=\sum_{i}a_{i}$	Total power allocated to all private streams
$a_{i}^{†}=\sum_{j\neq i}a_{j}$	Sum of all private-stream coefficients except *i*’s
*P*_*U*_	UAV transmit power
$s_{c},\;s_{i}$	Normalized (unit-power) common and private message streams
*y*_*i*_	Received signal at user *i*
$\omega_{i}$	AWGN at user *i*
$\sigma_{i}^{2},\;\sigma_{W}^{2}$	Noise variances at user *i* and Willie
$\rho_{U}$	Transmit SNR at the UAV
${\bar{\gamma }}_{c,i},\;{\bar{\gamma }}_{p,i}$	SINRs for decoding common and private streams at user *i*
${ℋ}_{0},{ℋ}_{1}$	Hypotheses of “no covert transmission” and “covert transmission”
*P*_*W*_	Average received power at Willie
*λ*	Willie’s detection threshold
$D_{0},\;D_{1}$	Willie’s decisions in favor of ${ℋ}_{0}$ or ${ℋ}_{1}$
$p_{f},\;p_{m}$	False-alarm and missed-detection probabilities
$\varepsilon_{th}^{c},\;\varepsilon_{th}^{p,i}$	SINR thresholds for common and private streams
κ	Rate-splitting distribution coefficient
Ei(.)	Exponential integral function
Γ(.)	Gamma function

In particular, we represent the coordinates of the *i*th user as (*x*_*i*_, *y*_*i*_, 0), the coordinates of Willie as (*x*_*W*_, *y*_*W*_, *h*_*W*_), and the coordinates of the UAV as (*x*_*U*_, *y*_*U*_, *h*_*U*_), where *h*_*U*_ signifies the altitude of the UAV. Consequently, the Euclidean distances between the *i*th user and the UAV, as well as between Willie and the UAV, are expressed as $d_{i}=\sqrt{h_{U}^{2}+r_{i}^{2}}$ and $d_{W}=\sqrt{h_{U}^{2}+r_{W}^{2}}$, respectively, with the elevation angle denoted as $\varphi_{a}=\tan^{-1}\left(\frac{h_{U}}{r_{a}}\right)$, a∈{i,W} in which $r_{i}=\sqrt{{\left(x_{U}-x_{i}\right)}^{2}+{\left(y_{U}-y_{i}\right)}^{2}}$ and $r_{W}=\sqrt{{\left(x_{U}-x_{W}\right)}^{2}+{\left(y_{U}-y_{W}\right)}^{2}}$ are the horizontal distance between the UAV to *i*th user and the UAV to Willie, respectively. We consider a probabilistic framework that accounts for both line-of-sight (LoS) and non-line-of-sight (NLoS) conditions. In this context, the large-scale fading characteristics of the channels between the UAV and both the *i*th user and Willie are explicitly modeled. This presumption integrates the probabilities associated with LoS and NLoS connections between the UAV and terrestrial devices to compute the average path loss as delineated in [43]

$$\begin{array}{cc} D_{a}=\left[N_{\text{NLoS}}+\frac{N_{\text{LoS}}-N_{\text{NLoS}}}{1+\beta e^{-\frac{180\alpha \varphi_{a}}{\pi }+\alpha \beta }}\right]d_{a}^{\delta } & ,a\in \left\{i,W\right\}, \end{array}$$ (1)

Here, *δ* represents the path-loss exponent, *α* and *β* are constants that are contingent upon the environmental context and N♢=E♢(c/cfc4πfc4π) −1 denotes parameters that are influenced by the surroundings, terrain, and carrier frequency, where ♢∈{LoS,NLoS}, *c* signifies the velocity of light in atmospheric conditions, *f*_*c*_ is the carrier frequency, and $E_{♢}$ denotes the additional path losses associated with both LoS and NLoS transmission modalities.

It is posited that all communication channels can be characterized as Nakagami-*m* fading channels, with the channel coefficients represented as random variables (RVs) that adhere to the Nakagami-*m* distribution model [44]. Consequently, the cumulative distribution function (CDF) and probability density function (PDF) of the respective channel power gain can be articulated as follows:

$$F_{{\left|h_{a}\right|}^{2}}\left(x\right)=1-e^{-\mu_{a}x}\sum_{p=0}^{m_{a}-1}\frac{\mu_{a}^{p}x^{p}}{p!},$$ (2a)

$$f_{{\left|h_{a}\right|}^{2}}\left(x\right)=\frac{\mu_{a}^{m_{a}}x^{m_{a}-1}}{\Gamma \left(m_{a}\right)}e^{-\mu_{a}x},$$ (2b)

where Γ(.) is the Gamma function, μa=ma/maΩaΩa in which $\Omega_{a}$ stands for the average channel power gain and *m*_*a*_ is the fading severity factor. For analytical simplicity, we assume homogeneous fading with $m_{W}=m_{i}=m,\forall i\in \left\{1,\ldots ,N\right\}$. However, we also evaluate heterogeneous scenarios in our simulations, where user fading remains fixed and *m*_*W*_ varies independently to reflect realistic channel diversity.

To provide a smooth transition to the RSMA-based communication model, we now define the transmit signal from the UAV. Under the RSMA protocol, the UAV transmits a superimposed signal containing a common stream shared by all users and multiple private streams for individual users. The transmit signal can be expressed as:

$$s=\sqrt{a_{c}P_{U}}s_{c}+\sum_{i=1}^{N}\sqrt{a_{i}P_{U}}s_{i},$$ (3)

where *P*_*U*_ denotes the total transmission power, *s*_*c*_ is the common message, and *s*_*i*_ is the private message for user *U*_*i*_. The coefficients *a*_*c*_ and *a*_*i*_ represent the power allocation ratios and satisfy $a_{c}+\sum_{i=1}^{N}a_{i}=1$.

### 2.2 Communication protocol

The UAV adopts the RSMA protocol, which separates transmitted content into a common part and multiple private parts. The common message *s*_*c*_ is encoded to be decodable by all users, while each *s*_*i*_ carries information targeted at user *U*_*i*_. The power allocation among these streams is controlled by coefficients *a*_*c*_ and *a*_*i*_, introduced in (3). The parameter *a*_*p*_ denotes the total power allocated to all private messages, i.e., $a_{p}=\sum_{i=1}^{N}a_{i}$, and $a_{i}^{†}=\sum_{j=1,j\neq i}^{N}a_{j}$ represents the power allocated to private messages for users other than *U*_*i*_.

The signal acquired at the *i*th user may be articulated as

$$y_{i}=\sqrt{D_{i}^{-1}}h_{i}s+\omega_{i}\;=\underset{\begin{array}{c} \text{Common}\;\text{message} \end{array}}{\underbrace{h_{i}\sqrt{a_{c}D_{i}^{-1}P_{U}}s_{c}}}+\underset{\begin{array}{c} \text{Desired}\;\text{private}\;\text{message} \end{array}}{\underbrace{h_{i}\sqrt{a_{i}D_{i}^{-1}P_{U}}s_{i}}}\;+\underset{\begin{array}{c} \text{Interfernce} \end{array}}{\underbrace{\sum_{j=1,j\neq i}^{N}\sqrt{a_{j}D_{i}^{-1}P_{U}}h_{i}s_{j}}}+\underset{\begin{array}{c} \text{AWGN}, \end{array}}{\underbrace{\omega_{i}}}$$ (4)

where $\omega_{i}∼CN\left(0,\sigma_{i}^{2}\right)$ is additive white Gaussian noise (AWGN) with zero mean and *N*_0_ variance.

In accordance with the RSMA protocol, the user initially decodes the common message *s*_*c*_ while considering the private message as a source of interference. Following this, the user engages in successive interference cancellation (SIC) to remove *s*_*c*_, subsequently allowing decoding its own private message stream, while considering the private message streams from other users as interference. Therefore, the signal-to-interference ratio (SINR) for decoding *s*_*c*_ and *s*_*i*_ at *U*_*i*_ can be articulated, respectively, as

$${\bar{\gamma }}_{c,i}=\frac{a_{c}D_{i}^{-1}P_{U}{\left|h_{i}\right|}^{2}}{a_{p}D_{i}^{-1}P_{U}{\left|h_{i}\right|}^{2}+N_{0}}=\frac{a_{c}\rho_{U}{\left|h_{i}\right|}^{2}}{a_{p}\rho_{U}{\left|h_{i}\right|}^{2}+D_{i}},$$ (5)

and

$${\bar{\gamma }}_{p,i}=\frac{a_{i}D_{i}^{-1}P_{U}{\left|h_{i}\right|}^{2}}{a_{i}^{†}D_{i}^{-1}P_{U}{\left|h_{i}\right|}^{2}+N_{0}}=\frac{a_{i}\rho_{U}{\left|h_{i}\right|}^{2}}{a_{i}^{†}\rho_{U}{\left|h_{i}\right|}^{2}+D_{i}},$$ (6)

where $\rho_{U}=P_{U}/\sigma_{i}^{2}$ is the transmit SNR. Note that *s*_*c*_ and *s*_*i*_ are expected to be normalized unity power signals, i.e., $𝔼\left\{s_{c}^{2}\right\}=𝔼\left\{s_{i}^{2}\right\}=1$ in which 𝔼{.} represents the mean operation.

Furthermore, Willie seeks to ascertain whether the UAV is emitting clandestine signals to User 1 based on the power of the signals received. The null hypothesis $H_{0}$ posits that the UAV is not engaging in transmission towards User 1, whereas the alternative hypothesis $H_{1}$ asserts that the BS is surreptitiously relaying information to User 1. Consequently, the signal received by Willie under the conditions of $H_{0}$ and $H_{1}$ can be expressed as

$${\bar{\gamma }}_{W}=\{\begin{array}{cc} {\left|h_{W}\right|}^{2}\sqrt{D_{W}^{-1}}\left(\sqrt{a_{c}P_{U}}s_{c}+\sum_{i=2}^{N}\sqrt{a_{i}P_{U}}s_{i}\right)+\omega_{W} & ,H_{0}\\ {\left|h_{W}\right|}^{2}\sqrt{D_{W}^{-1}}\left(\sqrt{a_{c}P_{U}}s_{c}+\sum_{i=1}^{N}\sqrt{a_{i}P_{U}}s_{i}\right)+\omega_{W} & ,H_{1} \end{array}$$ (7)

Here, $\omega_{W}∼CN\left(0,\sigma_{W}^{2}\right)$ represents the AWGN at Willie. According to (7), Willie’s average received power is

$$P_{W}=\{\begin{array}{ll} D_{W}^{-1}P_{U}{\left|h_{W}\right|}^{2}\left(a_{c}+a_{1}^{†}\right)+\sigma_{W}^{2}, & H_{0}\\ D_{W}^{-1}P_{U}{\left|h_{W}\right|}^{2}+\sigma_{W}^{2}, & H_{1} \end{array}$$ (8)

where $a_{1}^{†}$ is already given above (3), the decision rule is expressed as

$$P_{W}\overset{D_{1}}{\underset{D_{0}}{≷}}\lambda ,$$ (9)

where *λ* is Willie’s power detection threshold, *D*_1_ and *D*_0_ are the respective decisions in favor of $H_{1}$ and $H_{0}$.

## 3 Performance analysis

In this segment, we investigate the efficacy of clandestine communication within multi-user communication frameworks supported by UAV. Specifically, we initially formulate a precise mathematical representation for the DEP at Willie, and subsequently examine the probability of user outage alongside the expression for the covert transmission rate resolution.

### 3.1 Detection error probability

In this subsection, we examine the likelihood that Willie is able to accurately identify the clandestine communication transmitted by the UAV to the covert User 1. More precisely, Willie must discern whether the UAV conveys a covert signal under the hypotheses $H_{0}$ and $H_{1}$, which subsequently yields the binary detection outcomes *D*_0_ and *D*_1_. We employ the DEP metric to quantify Willie’s detection efficacy, as referenced in [45]. The DEP can be articulated as

$$\text{DEP = Pr}\left[D_{1}|H_{0}\right]+\text{Pr}\left[D_{0}|H_{1}\right],$$ (10)

where $p_{f}\overset{\Delta }{=}\text{Pr}\left[D_{1}|H_{0}\right]=\text{Pr}\left[P_{W}>\lambda |H_{0}\right]$ denotes the *probability of a false alarm*, which refers to the identification of a covert signal in the absence of any transmitted information, and $p_{m}\overset{\Delta }{=}\text{Pr}\left[D_{0}|H_{1}\right]=\text{Pr}\left[P_{W}<\lambda |H_{1}\right]$ signifies the *probability of a missed detection*, indicating the failure to identify a covert signal when information is indeed transmitted. Specifically, a DEP value of 0 signifies that Willie is capable of flawlessly identifying the clandestine signal transmitted from the UAV to User 1, devoid of any errors, whereas a DEP value of 1 indicates that Willie is incapable of detecting the covert signal.

According to Eqs (8) and (10), the likelihood of false alarms (*p*_*f*_) and the probability of missed detections (*p*_*m*_) at Willie can be expressed as

$$p_{f}=\text{Pr}\left[P_{W}>\lambda |H_{0}\right]\;=\{\begin{array}{cc} 1, & \lambda \leq \sigma_{W}^{2}\\ e^{-\frac{\mu_{W}\Theta }{D_{W}^{-1}P_{U}\left(a_{c}+a_{1}^{†}\right)}}\sum_{p=0}^{m_{W}-1}\frac{\mu_{W}^{p}\Theta^{p}}{p!{\left[D_{W}^{-1}P_{U}\left(a_{c}+a_{1}^{†}\right)\right]}^{p}}, & \lambda >\sigma_{W}^{2} \end{array}$$ (11)

and

$$p_{m}=\text{Pr}\left[P_{W}<\lambda |H_{1}\right]\;=\{\begin{array}{cc} 1, & \lambda \leq \sigma_{W}^{2}\\ 1-e^{-\frac{\mu_{W}\Theta }{D_{W}^{-1}P_{U}}}\sum_{p=0}^{m_{W}-1}\frac{\mu_{W}^{p}\Theta^{p}}{p!{\left[D_{W}^{-1}P_{U}\right]}^{p}}, & \lambda >\sigma_{W}^{2} \end{array}$$ (12)

where $\Theta =\lambda -\sigma_{W}^{2}$.

Plugging (12) and (11) into (10) and performing a series of algebraic manipulations, the closed-form expression for DEP at Willie is represented as

$$\text{DEP }=p_{f}+p_{m}\;=\text{Pr}\left[P_{W}>\lambda |H_{0}\right]+\text{Pr}\left[P_{W}<\lambda |H_{1}\right]\;=\{\begin{array}{ll} 1, & \lambda \leq \sigma_{W}^{2}\\ 1+\sum_{p=0}^{m_{W}-1}\frac{\mu_{W}^{p}\Theta^{p}e^{-\frac{\mu_{W}\Theta }{D_{W}^{-1}P_{U}\left(a_{c}+a_{1}^{†}\right)}}}{p!{\left[D_{W}^{-1}P_{U}\left(a_{c}+a_{1}^{†}\right)\right]}^{p}}-\sum_{p=0}^{m_{W}-1}\frac{\mu_{W}^{p}\Theta^{p}e^{-\frac{\mu_{W}\Theta }{D_{W}^{-1}P_{U}}}}{p!{\left[D_{W}^{-1}P_{U}\right]}^{p}}, & \lambda >\sigma_{W}^{2}, \end{array}\;=\{\begin{array}{ll} 1 & \lambda \leq \sigma_{W}^{2}\\ 1+\sum_{p=0}^{m_{W}-1}\frac{\mu_{W}^{p}D_{W}^{p}\Theta^{p}}{p!}\left[\frac{e^{-\frac{\mu_{W}D_{W}\Theta }{P_{U}\left(a_{c}+a_{1}^{†}\right)}}}{P_{U}^{p}{\left(a_{c}+a_{1}^{†}\right)}^{p}}-\frac{e^{-\frac{\mu_{W}D_{W}\Theta }{P_{U}}}}{P_{U}^{p}}\right], & \lambda >\sigma_{W}^{2} \end{array}$$ (13)

**Remark 1.** Let us analyze the case where *a*_*c*_ is 0. As can be seen, the UAV no longer transmits common message streams to the users, thus transforming the system from an RSMA covert transmission framework to a NOMA covert transmission framework. Now, $\mu_{W}D_{W}\Theta /P_{U}\left(a_{c}+a_{1}^{†}\right)$ given in (11) becomes smaller, while μWDWΘ/μWDWΘPUPU given in (12) remains the same. Consequently, *p*_*m*_ remains at the same level, but the value of *p*_*f*_ decreases, according to the properties of the exponential function. Furthermore, since DEP is represented as the summation of *p*_*f*_ and *p*_*m*_, it can be concluded that the DEP of the NOMA system is inferior to that of the RSMA system. This finding corroborates the conclusion that the implementation of the RSMA system enhances the efficiency of covert communication.

**Remark 2.** In high SNR conditions, as indicated by Eqs (12) and (11), it is evident that $p_{m}\to 0$ and $p_{f}\to 1$. Consequently, since $\text{DEP = }p_{f}+p_{m}$, it follows that DEP→1, which signifies that covert transmission is guaranteed in elevated SNR scenarios.

### 3.2 UAV trajectory optimization via alternating optimization

While a full trajectory optimization over time is crucial for mobile UAV systems, it presents significant complexity and is considered beyond the scope of this paper. To address the reviewer’s concern in a foundational manner, we first tackle the problem of optimizing the UAV’s **static position**. This provides crucial insights into how spatial parameters affect system covertness and serves as a foundational step for future studies on dynamic trajectories. Specifically, to enhance the covertness of the UAV-assisted RSMA system, we propose an alternating optimization (AO)-based algorithm. The objective is to determine the optimal 3D position of the UAV that maximizes the average DEP at Willie, while maintaining communication requirements. The proposed method iteratively optimizes each spatial coordinate (*x*_*U*_, *y*_*U*_, *h*_*U*_) while holding the others fixed. This approach offers a favorable trade-off between computational complexity and covert performance.

**Algorithm 1 AO-based UAV trajectory optimization for covert RSMA transmission [46].**

1 Initialize the search ranges: $x=x_{\min}:1:x_{\max}$, $y=y_{\min}:1:y_{\max}$, $h=h_{\min}:1:h_{\max}$; maximum iterations $n_{\max}$; set *n* = 1.

2 Randomly select initial UAV position: *x*_*U*_, *y*_*U*_, *h*_*U*_.

3 Compute initial DEP: $\tilde{\text{ DEP}}(1)=\text{ DEP}(x_{U},y_{U},h_{U})$.

4 **Repeat:**

• For all $x_{i}\in x$, compute: ${\text{temp}}_{x}(i)=\tilde{\text{ DEP}}(x_{i},y_{U},h_{U})$.

• Update: $x_{ao}={\mathrm{arg max}}_{x}({\text{temp}}_{x})$.

• For all $y_{j}\in y$, compute: ${\text{temp}}_{y}(j)=\tilde{\text{ DEP}}(x_{ao},y_{j},h_{U})$.

• Update: $y_{ao}={\mathrm{arg max}}_{y}({\text{temp}}_{y})$.

• For all $z_{k}\in z$, compute: ${\text{temp}}_{z}(k)=\tilde{\text{ DEP}}(x_{ao},y_{ao},h_{k})$.

• Update: $z_{ao}={\mathrm{arg max}}_{z}({\text{temp}}_{z})$.

Compute updated DEP: $\tilde{\text{ DEP}}(n+1)=\text{ DEP}(x_{ao},y_{ao},h_{ao})$.

**If** $\tilde{\text{ DEP}}(n+1)=\tilde{\text{ DEP}}(n)$ or $n=n_{\max}$: **Stop**.

**Else**: update UAV position: $x_{U}=x_{ao}$, $y_{U}=y_{ao}$, $h_{U}=h_{ao}$,

and set

*n* = *n* + 1.

**End Repeat**

**Output:** Optimal position of UAV: $\left(x_{U},y_{U},h_{U})=(x_{ao},y_{ao},h_{ao}\right)$.

The computational complexity of Algorithm 3.1 is $𝒪(n_{\max}(L_{x}+L_{y}+L_{z}))$, where 𝒪 denotes Big 𝒪 notation, *L*_*x*_, *L*_*y*_, and *L*_*z*_ denote the number of grid points along each axis. Compared to exhaustive 3D search, this method significantly reduces runtime while maintaining near-optimal covert performance.

### 3.3 Outage probability

For the link between the UAV and a user in this RSMA system to operate successfully (non-outage), the user must correctly decode both the common and private messages. This necessity arises because the UAV sends a combined signal containing both message types, and users employ a two-step decoding procedure: decoding the common message first, then using SIC to decode their private message. Broadly speaking, an outage event will happen with a certain probability if the SINR for decoding either the common message (${\bar{\gamma }}_{c,i}$) or the private message (${\bar{\gamma }}_{p,i}$) is lower than its respective threshold. Consequently, the outage probability with *U*_*i*_ can be articulated as

$$O_{U_{i}}=1-\mathrm{Pr}\left({\bar{\gamma }}_{c,i}>\varepsilon_{th}^{c},{\bar{\gamma }}_{p,i}>\varepsilon_{th}^{p,i}\right),$$ (14)

Here, $\varepsilon_{th}^{c}$ and $\varepsilon_{th}^{p,i}$ represent the threshold SINRs for the common and private messages, respectively.

With the help of (2a) and after some algebraic simplifications, the outage probability at the *i*th user is given by

$${𝒪}_{U_{i}}=\left\{ \begin{array}{cc} 1-e^{-\frac{\mu_{i}{𝒟}_{i}\varepsilon_{th}^{p,i}}{\rho_{U}(a_{i}-\varepsilon_{th}^{p,i}a_{i}^{†})}}\sum_{p=0}^{m_{i}-1}\frac{1}{p!}{\left[\frac{\mu_{i}^{p}{𝒟}_{i}\varepsilon_{th}^{p,i}}{\rho_{U}(a_{i}-\varepsilon_{th}^{p,i}a_{i}^{†})}\right]}^{p}, & \text{if }\varepsilon_{th}^{c}<\varepsilon_{th}^{p,i}\\ 1-e^{-\frac{\mu_{i}{𝒟}_{i}\varepsilon_{th}^{c}}{\rho_{U}(a_{c}-\varepsilon_{th}^{c}a_{p})}}\sum_{p=0}^{m_{i}-1}\frac{1}{p!}{\left[\frac{\mu_{i}^{p}{𝒟}_{i}\varepsilon_{th}^{c}}{\rho_{U}(a_{c}-\varepsilon_{th}^{c}a_{p})}\right]}^{p}, & \text{if }\varepsilon_{th}^{c}\geq \varepsilon_{th}^{p,i} \end{array}\right.$$ (15)

In order to obtain more useful insights, we perform an asymptotic analysis of OP. We start with the asymptotic of the PDF of the Nakagami-*m* distribution when $\rho_{U}\to \infty$ as follows [49]

$$F_{{\left|h_{a}\right|}^{2}}^{\infty }(x)\approx \frac{\mu_{a}^{m_{a}}x^{m_{a}}}{\Gamma \left(m_{g}+1\right)}.$$ (16)

Submitting (16) into (15), the asymptotic expression for the OP of *i*th user is derived as

$${𝒪}_{U_{i}}^{\infty }=\left\{ \begin{array}{cc} \frac{1}{\Gamma (m_{i}+1)}{\left[\frac{\mu_{i}{𝒟}_{i}\varepsilon_{th}^{p,i}}{\rho_{U}(a_{i}-\varepsilon_{th}^{p,i}a_{i}^{†})}\right]}^{m_{i}}, & \text{if }\varepsilon_{th}^{c}<\varepsilon_{th}^{p,i}\\ \frac{1}{\Gamma (m_{i}+1)}{\left[\frac{\mu_{i}{𝒟}_{i}\varepsilon_{th}^{c}}{\rho_{U}(a_{c}-\varepsilon_{th}^{c}a_{p})}\right]}^{m_{i}}, & \text{if }\varepsilon_{th}^{c}\geq \varepsilon_{th}^{p,i} \end{array}\right.$$ (17)

It is evident that the OP shows an upward trend in correlation with the increase in the SINR threshold and shows a downward trend as *P*_*U*_ increases. Consequently, one can mitigate the OP of the system by lowering the threshold SINR and enhancing *P*_*U*_.

**Remark 3.** From the definition of the diversity order, which is defined as ${𝔻}_{U_{i}}=-\underset{\rho_{U}\to \infty }{\lim}\frac{\log\left(O_{U_{i}}^{\infty }\left(\rho_{U}\right)\right)}{\log\left(\rho_{U}\right)}$, when $\rho_{U}$ goes to infinity, the diversity order achieved by the *i*th user is *m*_*i*_.

**Remark 4.** Examine the scenario where *a*_*c*_ = 0. In this instance, the system is streamlined to function as a NOMA framework. It can be discerned that within the NOMA configuration, the outage probability is represented as $O_{U_{i}}^{NOMA}=1-e^{-\frac{\mu_{i}D_{i}\varepsilon_{th}^{p,i}}{\rho_{U}\left(a_{i}-\varepsilon_{th}^{p,i}a_{i}^{†}\right)}}\sum_{p=0}^{m_{i}-1}\frac{\mu_{i}^{p}}{p!}{\left[\frac{D_{i}\varepsilon_{th}^{p,i}}{\rho_{U}\left(a_{i}-\varepsilon_{th}^{p,i}a_{i}^{†}\right)}\right]}^{p}$. When juxtaposed with the RSMA system, the outage probability of the NOMA system is at least equivalent to, if not more favorable than, that of the RSMA system. Nonetheless, in the context of this covert communication system, the metric of covertness is regarded as a more advantageous performance criterion than the outage probability. As articulated in Remark 1, the covertness capabilities of the RSMA system surpass those of the NOMA system. Consequently, the utilization of the RSMA system in this scenario emerges as a more advantageous option.

### 3.4 Ergodic rate analysis

Beyond outage probability and covertness detection metrics, the achievable data rates represent fundamental performance measures for the communication system. In this section, we analyze the ergodic rates for the *i*th user (*U*_*i*_) in the considered UAV-assisted RSMA network. Ergodic rates quantify the long-term average data transmission rates achievable over the fading channel conditions. Following the methodology in [50] and utilizing the SINR expressions for the common stream (${\bar{\gamma }}_{c,i}$) and private stream (${\bar{\gamma }}_{p,i}$) given in (5) and (6), respectively. The ergodic rate for *U*_*i*_ decoding the common information stream *s*_*c*_ is defined as the expectation of the instantaneous rate:

$$R_{c,i}=𝔼\left[\log_{2}\left(1+{\bar{\gamma }}_{c,i}\right)\right].$$ (18)

Similarly, the ergodic rate for *U*_*i*_ successfully decoding its private information stream *s*_*i*_ after applying SIC is given by

$$R_{p,i}=𝔼\left[\log_{2}\left(1+{\bar{\gamma }}_{p,i}\right)\right].$$ (19)

It is essential for the RSMA protocol that all users can decode the common stream. Therefore, the overall common rate *R*_*c*_ that can be reliably transmitted is limited by the user experiencing the minimum rate, i.e., $R_{c}=\min\left(R_{c,1},R_{c,2},\ldots ,R_{c,N}\right)$. Furthermore, because covert User 1 and other public users share the transmission rate of the entire common information, we assume that their common transmission rates are $C_{c,1}$ and $\sum_{i=2}^{N}C_{c,i}$, respectively, and we may calculate $C_{c,1}+\sum_{i=2}^{N}C_{c,i}=R_{c}$. Based on the analysis, the ergodic rate may be expressed as

$$C_{R,i}=\kappa R_{c}+R_{p,i}$$ (20)

Here, κ is the coefficient of the rate distribution satisfying the inequality 0<κ<1. Then, *R*_*c*,*i*_ is given as

$$R_{c,i}=\frac{1}{\ln2}\sum_{p=0}^{m_{i}-1}\frac{\mu_{i}^{p}D_{i}^{p}}{p!}\langle \left[{\left(-1\right)}^{p-1}\psi_{1}^{p}e^{D_{i}\mu_{i}\psi_{1}}\text{Ei}\left(-D_{i}\mu_{i}\psi_{1}\right)+\sum_{r=1}^{p}\frac{\left(r-1\right)!{\left(-\psi_{1}\right)}^{p-r}}{{\left(D_{i}\mu_{i}\right)}^{r}}\right]\;-\left[{\left(-1\right)}^{p-1}\psi_{2}^{p}e^{D_{i}\mu_{i}\psi_{2}}\text{Ei}\left(-D_{i}\mu_{i}\psi_{2}\right)+\sum_{r=1}^{p}\frac{\left(r-1\right)!{\left(-\psi_{2}\right)}^{p-r}}{{\left(D_{i}\mu_{i}\right)}^{r}}\right]\rangle ,$$ (21)

where Ei(.) is the exponential integral function, $\psi_{1}={\left[\rho_{U}\left(a_{c}+a_{p}\right)\right]}^{-1}$ and $\psi_{2}=\left(\rho_{U}a_{p}\right){\;\;}^{-1}$.

*Proof:* We can rewrite *R*_*c*,*i*_ as follows

$$R_{c,i}=𝔼\left[\log_{2}\left(1+{\bar{\gamma }}_{c,i}\right)\right]=\frac{1}{\ln2}\int_{0}^{\frac{a_{c}}{a_{p}}}\frac{1}{1+x}\left[1-F_{{\left|h_{i}\right|}^{2}}\left(\frac{D_{i}x}{\rho_{U}\left(a_{c}-xa_{p}\right)}\right)\right]dx.$$ (22)

By the variable changing t=x/xρU(ac−xap)ρU(ac−xap) and with the help of [51], *R*_*c*,*i*_ can be determined by

$$R_{c,i}=\frac{1}{\ln2}\int_{0}^{\infty }\left(\frac{1}{t+\psi_{1}}-\frac{1}{t+\psi_{2}}\right)\left[1-F_{{\left|h_{i}\right|}^{2}}\left(D_{i}t\right)\right]dt,$$ (23)

where $\psi_{1}={\left[\rho_{U}\left(a_{c}+a_{p}\right)\right]}^{-1}$ and $\psi_{2}=\left(\rho_{U}a_{p}\right){\;\;}^{-1}$. Plugging (2a) into (23), we obtain

$$R_{c,i}=\frac{1}{\ln2}\sum_{p=0}^{m_{i}-1}\frac{\mu_{i}^{p}D_{i}^{p}}{p!}\int_{0}^{\infty }\left(\frac{1}{t+\psi_{1}}-\frac{1}{t+\psi_{2}}\right)e^{-D_{i}\mu_{i}t}t^{p}dt\;=\frac{1}{\ln2}\sum_{p=0}^{m_{i}-1}\frac{\mu_{i}^{p}D_{i}^{p}}{p!}\left\langle \int_{0}^{\infty }\frac{e^{-D_{i}\mu_{i}t}t^{p}}{t+\psi_{1}}dt-\int_{0}^{\infty }\frac{e^{-D_{i}\mu_{i}t}t^{p}}{t+\psi_{2}}dt\right\rangle$$ (24)

Solving the above using [52, Eq (3.353.5)], and we can obtain (21) after some algebraic simplifications. The proof is completed.

Similarly, using (6), the ergodic capacity of the private message can be expressed as $R_{p,i}=𝔼\left[\log_{2}\left(1+{\bar{\gamma }}_{p,i}\right)\right]$. Solving *R*_*c*,*i*_, using steps similar to (22), we obtain

$$R_{p,i}=\frac{1}{\ln2}\sum_{p=0}^{m_{i}-1}\frac{\mu_{i}^{p}D_{i}^{p}}{p!}\langle \left[{\left(-1\right)}^{p-1}\psi_{3}^{p}e^{D_{i}\mu_{i}\psi_{3}}\text{Ei}\left(-D_{i}\mu_{i}\psi_{3}\right)+\sum_{r=1}^{p}\frac{\left(r-1\right)!{\left(-\psi_{3}\right)}^{p-r}}{{\left(D_{i}\mu_{i}\right)}^{r}}\right]\;-\left[{\left(-1\right)}^{p-1}\psi_{4}^{p}e^{D_{i}\mu_{i}\psi_{4}}\text{Ei}\left(-D_{i}\mu_{i}\psi_{4}\right)+\sum_{r=1}^{p}\frac{\left(r-1\right)!{\left(-\psi_{4}\right)}^{p-r}}{{\left(D_{i}\mu_{i}\right)}^{r}}\right]\rangle ,$$ (25)

where $\psi_{3}={\left[\rho_{U}\left(a_{i}+a_{i}^{†}\right)\right]}^{-1}$ and $\psi_{4}=\left(\rho_{U}a_{i}^{†}\right){\;\;}^{-1}$.

Using (25) and (21) into (20), we obtain the ergodic rate for *U*_*i*_ is given by

$$C_{R,i}=\min\left(R_{c,1},R_{c,2},\ldots ,R_{c,N}\right)\kappa +R_{p,i}.$$ (26)

*High SNR Region*: As the transmit SNR becomes large ($\rho_{U}\to \infty$), the instantaneous SINRs converge towards constants determined by power allocation: ${\tilde{\gamma }}_{c,n}^{\infty }\to \frac{a_{c}}{a_{p}}$ and ${\tilde{\gamma }}_{p,n}^{\infty }\to \frac{a_{i}}{a_{i}^{†}}$. The corresponding asymptotic ergodic rates at *U*_*i*_ can be approximated as

$$R_{c,i}^{\infty }≃\log_{2}\left(1+\frac{a_{c}}{a_{p}}\right),$$ (27a)

$$R_{p,i}^{\infty }≃\log_{2}\left(1+\frac{a_{i}}{a_{i}^{†}}\right),$$ (27b)

From (27a) and (27b), the ergodic rate under high SNR can be expressed as

$$C_{R_{i}}^{\infty }=\min\left(R_{c,1}^{\infty },R_{c,2}^{\infty },\ldots ,R_{c,N}^{\infty }\right)\kappa +R_{p,i}.$$ (28)

These asymptotic values provide insights into the maximum achievable rates limited by interference in the high-power regime.

## 4 Numerical results

In this segment, we substantiate the findings through Monte Carlo simulations to verify the accuracy of the mathematical expressions. The system parameters are delineated in Table 2. Our simulations are predicated on link-level modeling. In particular, we generate 10^6^ stochastic channels to emulate authentic communication settings. Without loss of generality, we assume *N* = 2, a1=a2=(1−ac)/(1−ac)NN and $m=m_{W}=m_{i}$. The fading parameter *m* is varied as m = 1,2,3,4 to capture different wireless propagation conditions. Specifically, *m* = 1 corresponds to Rayleigh-like severe fading, while *m* = 4 reflects near-LoS scenarios with minimal multipath. This range is selected to emulate realistic UAV communication environments, from harsh urban deployments to more favorable open-air conditions.

**Table 2:** Simulation parameter [47,48].

Parameter	Value	Parameter	Value
$\left(x_{W},y_{W}\right)$	(15,0) (m)	(α,β)	(0.1581,9.6177)
$\left(x_{1},y_{1}\right)$	(5,0) (m)	$\left({ℰ}_{\text{LOS}},{ℰ}_{\text{NLOS}}\right)$	(1,20)
$\left(x_{2},y_{2}\right)$	(15,0) (m)	$\left(\sigma_{W}^{2},\sigma_{i}^{2}\right)$	–30 (dBm)
*x*_*U*_	0 (m)	$\left(\Omega_{W},\Omega_{i}\right)$	(1,0.5)
*y*_*U*_	0 (m)	*f*_*c*_	10^6^ (Hz)
*h*_*U*_	20 (m)	*c*	3.10^8^ (m/s)
*m*	[1,…,4]	*a*_*c*_	0.2
κ	0.7	*δ*	2

**Fig 2:**
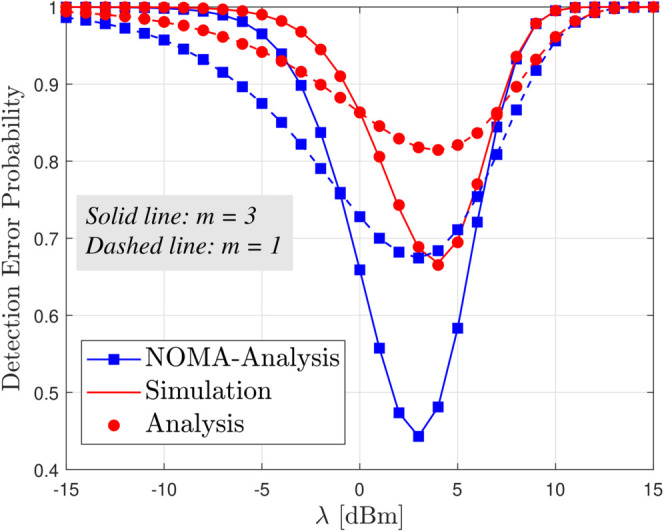
DEP versus threshold *λ* for different values of m, with *P*_*U*_ = 20 dBm.

Fig 2 shows the DEP at Willie versus the detection threshold *λ* under a fixed UAV transmit power of *P*_*U*_ = 20 dBm, comparing Nakagami-*m* fading conditions for *m* = 1 and *m* = 3, it is evident that both extremely low and high threshold values degrade detection performance low thresholds lead to frequent false alarms. In contrast, high thresholds cause many missed detections, pushing DEP toward 1. Yet, the optimal threshold yields a notably higher minimum DEP for *m* = 3 than for *m* = 1, indicating that while milder fading is typically advantageous in conventional communications, it enhances covertness by making it more difficult for Willie to discern the covert signal from noise reliably. Additionally, the DEP observed in NOMA systems is smaller than that in RSMA for small *λ* values, thus corroborating the findings noted in Remark 1.

**Fig 3:**
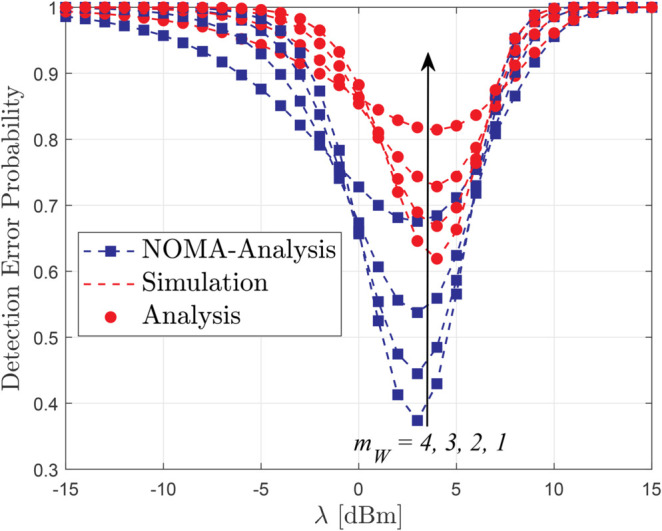
DEP versus threshold *λ* for different values of *m*_*w*_, with *m*_*i*_ = 2 and *P*_*U*_ = 20 dBm.

Fig 3 illustrates the impact of heterogeneous fading on the DEP when the legitimate users experience a fixed fading condition with *m*_*i*_ = 2, while the warden’s fading severity parameter *m*_*W*_ varies from 1 to 3. As expected, a smaller value of *m*_*W*_ (i.e., a more severe fading for Willie) leads to a higher DEP, indicating better covertness. In contrast, as *m*_*W*_ increases, the warden’s ability to detect covert transmissions improves, resulting in lower DEP. Nonetheless, across all considered values of *m*_*W*_, the RSMA-based covert communication maintains a considerable level of DEP, confirming the robustness of our proposed scheme against channel asymmetry.

**Fig 4:**
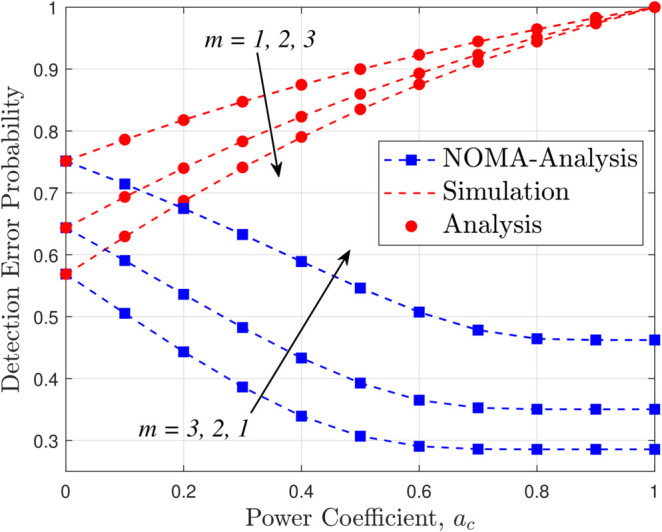
DEP versus power coefficient (*a*_*c*_), with m=1,2,3 and *P*_*U*_ = 20 dBm.

Fig 4 presents the DEP at Willie versus the power allocation coefficient *a*_*c*_ for different fading severities (*m* = 1, 2, and 3) under a fixed UAV transmit power of 20 dBm, and it is clear that variations in *a*_*c*_ significantly affect the allocation of power between common and private message streams with lower values of *a*_*c*_ generally favoring covert communications by reducing the power allocated to the common stream, thereby lowering Willie’s detection capability, while higher values increase the likelihood of detection, and the trends for different *m* values illustrate that as the fading becomes less severe (higher *m*), the minimum achievable DEP is higher—suggesting that channels with less severe fading, despite being preferable in conventional communications, actually enhance covert transmission by making it more challenging for Willie to reliably differentiate covert signals from noise—and, compared to NOMA systems where the DEP is smaller at low *a*_*c*_, these results further substantiate the covertness advantages of RSMA as highlighted in Remark 1.

**Fig 5:**
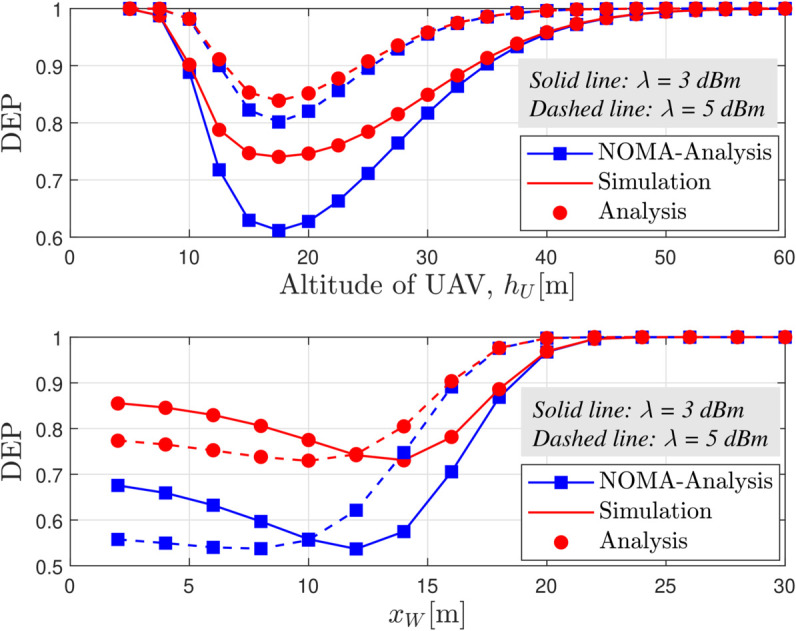
DEP versus *h*_*U*_ and *x*_*W*_ for different values of *λ*, with *m* = 2 and *P*_*U*_ = 18 dBm.

Fig 5 depicts the DEP at Willie as a function of the UAV altitude (*h*_*U*_) and Willie’s horizontal distance (*x*_*W*_) for various detection thresholds *λ* under a moderate fading condition (*m* = 2) and a UAV transmit power of *P*_*U*_ = 18 dBm, clearly illustrating that changes in *h*_*U*_ and *x*_*W*_ significantly influence Willie’s detection performance, while the comparison between RSMA and NOMA systems indicates that, although NOMA tends to achieve lower DEP at lower threshold settings, RSMA offers enhanced covertness by intelligently allocating power between the common and private message streams, thereby making it fundamentally more difficult for Willie to discern the covert transmission regardless of the variations in UAV altitude and Willie’s location. Moreover, the figure visually confirms the existence of a distinct optimal UAV position that maximizes DEP. This optimal value can be effectively determined by the proposed AO-based algorithm (as detailed in Algorithm 3.1), which provides a computationally efficient alternative to an exhaustive 3D search.

**Fig 6:**
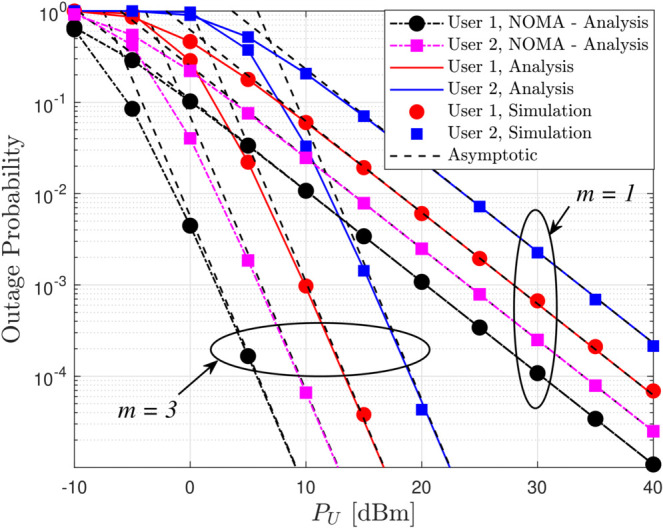
OP versus *P*_*U*_ for different values of *m* with *a*_*c*_ = 0.1, *x*_1_ = 15 m, *x*_2_ = 25 m, $\varepsilon_{th}^{c}=\varepsilon_{th}^{p,i}$ = -20 dBm.

Fig 6 shows the OP versus the UAV transmit power (*P*_*U*_) for different values of the fading severity parameter *m*, with system parameters set as *a*_*c*_ = 0.1, *x*_1_ = 15 m, *x*_2_ = 25 m, and SINR thresholds fixed at –20 dBm, clearly revealing that as *P*_*U*_ increases, the OP decreases and asymptotically approaches levels determined by the diversity order (equal to *m*), so that milder fading (higher m) yields a steeper decline in OP, and when comparing RSMA with NOMA systems, although NOMA may exhibit slightly lower OP in certain regions, RSMA’s strategic power allocation between common and private message streams not only enhances covert communication by making detection more difficult but also demonstrates robust asymptotic performance in high SNR scenarios, thereby affirming its overall advantage despite a potential trade-off with OP. This is in line with the conclusion stated in Remark 4.

**Fig 7:**
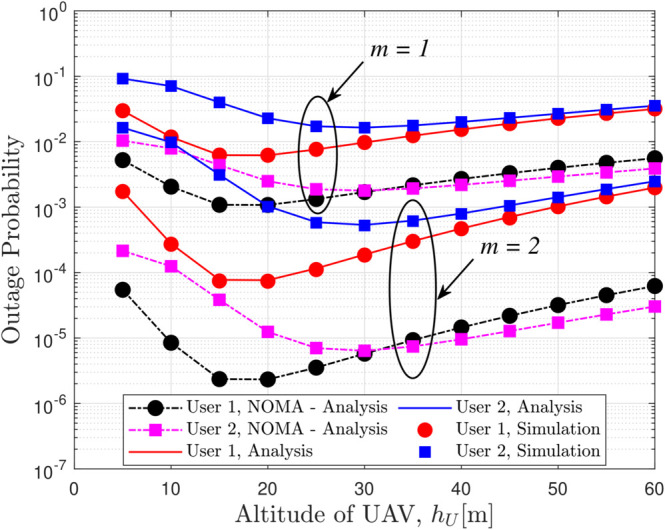
OP versus *h*_*U*_ for different values of *m* with *a*_*c*_ = 0.1, *x*_1_ = 15 m, *x*_2_ = 25 m, $\varepsilon_{th}^{c}=\varepsilon_{th}^{p,i}$ = -20 dBm.

Fig 7 depicts the OP versus the UAV altitude (*h*_*U*_) for different fading parameters (m) with system settings of *a*_*c*_ = 0.1, *x*_1_ = 15 m, *x*_2_ = 25 m, and SINR thresholds at *P*_*U*_ = −20 dBm, clearly demonstrating that as *h*_*U*_ increases the OP decreases, approaching an asymptotic behavior determined by the diversity order equal to *m* so that channels with milder fading exhibit a more pronounced reduction in OP and while NOMA may occasionally offer slightly lower OP values, the RSMA scheme, through its intelligent power allocation between common and private messages, ensures enhanced covert communication performance, thereby presenting a more favorable trade-off between outage performance and covertness.

**Fig 8:**
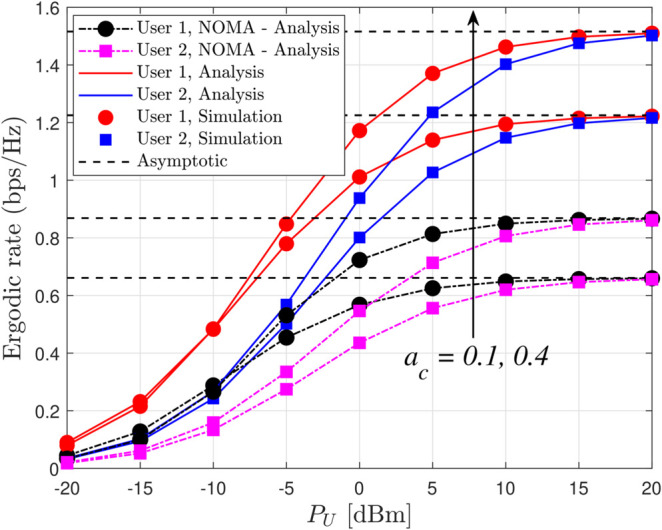
Ergodic rate versus *P*_*U*_ for different values of *a*_*c*_ with *x*_1_ = 15 m, *x*_2_ = 25 m, κ=0.7 and *m* = 2.

Fig 8 presents the ergodic rate versus the UAV transmit power (*P*_*U*_) for various power allocation coefficients ac with *x*_1_ = 15 m, *x*_2_ = 25 m, κ=0.7, and *m* = 2, clearly demonstrating that as *P*_*U*_ increases the ergodic rate improves, with the variation in ac affecting the balance between common and private message rates; notably, while conventional NOMA systems might achieve slightly lower ergodic rates under similar conditions, the RSMA framework by virtue of its flexible power allocation enhances covert communication performance and offers superior resilience in high SNR regimes, thereby solidifying its advantage in achieving better overall throughput and covertness.

**Fig 9:**
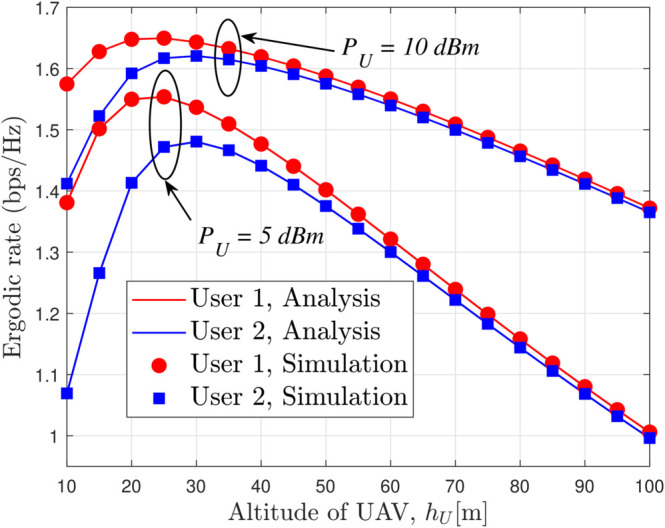
Ergodic rate versus *h*_*U*_ for different values of *P*_*U*_ with *x*_1_ = 15 m, *x*_2_ = 25 m, *a*_*c*_ = 0.5, κ=0.7 and *m* = 4.

Fig 9 illustrates the ergodic rate as a function of UAV altitude (*h*_*U*_) for different values of *P*_*U*_ with *x*_1_ = 15 m, *x*_2_ = 25 m, *a*_*c*_ = 0.5, κ=0.7, and *m* = 4, clearly demonstrating that an increase in UAV altitude leads to improved ergodic rates due to better channel conditions, while higher transmit power further boosts the overall performance; notably, the flexible power allocation in RSMA enables a more effective balance between common and private message rates compared to NOMA systems, thereby enhancing both throughput and covert communication efficacy, particularly in high SNR regimes where the RSMA scheme consistently outperforms NOMA by yielding higher ergodic rates and a more robust system behavior.

## 5 Conclusion

In this paper, we investigated the performance of covert communication in a multi-user UAV-aided network with RSMA. We considered the downlink scenario when the transmission to legitimate users, including a given covert user, is performed over Nakagami-*m* fading channels while Willie is suspected to be detected. We derive closed-form expressions for DEP, OP, and ER, where the DEP is utilized to quantify the warden’s ability to detect the presence or absence of communication activity between the UAV and the covert user. Asymptotic analysis further illuminated outage behavior in the high SNR regime. Numerical simulations validated our analytical model and exhibited the effect of significant system parameters, e.g., power allocation, altitude of UAV, and fading severity. Of specific interest, it was demonstrated that RSMA can offer significant covertness gains over a NOMA baseline, with greater minimum DEP, though perhaps at the expense of a trade-off with user outage probability. Our findings identify the potential of RSMA as an effective tool for secure UAV communications. Potential future work could extend the static UAV position optimization presented in this paper to a full dynamic trajectory design, further enhancing covertness in mobile scenarios. Additionally, analysis with multi-antenna configurations is another promising direction.
